# An Investigation into the Biological Activities of Four Lamiaceae Essential Oils Against *Thrips flavus*, Crops, and Weeds

**DOI:** 10.3390/plants14030448

**Published:** 2025-02-03

**Authors:** Yuxin Zhou, Tianhao Pei, Xuechao Zhou, Meng-Lei Xu, Hexin Gao, Lulu Wang, Yu Gao

**Affiliations:** 1 Key Laboratory of Soybean Disease and Pest Control, College of Plant Protection, Jilin Agricultural University, Ministry of Agriculture and Rural Affairs, Changchun 130118, China; 2 Chifeng Agricultural and Animal Husbandry Scientific Research Institute, Chifeng 024050, China; 3 State Key Laboratory of Supramolecular Structure and Materials, College of Food Science and Engineering, Jilin University, Changchun 130062, China; 4 Suzhou Academy of Agricultural Sciences, Suzhou 234000, China

**Keywords:** *Thrips flavus*, Lamiaceae, essential oil, *Glycine max*, *Zea mays*, weeds

## Abstract

In recent years, with the increasing awareness of environmental protection and food safety, essential oils (EOs) have gained significant attention as safer and more environmentally friendly alternatives. This study investigated the insecticidal activity of four Lamiaceae EOs (patchouli oil, catnip oil, lavender oil, and mint oil) against *Thrips flavus* and their effects on crops and weeds. The results show that patchouli oil, catnip oil, and lavender oil exhibited better insecticidal activity, with patchouli oil having the strongest toxicity, with an LC_50_ value of 0.31 mg/mL. Additionally, catnip oil and lavender oil had significant attractive effects on *T. flavus*, where lavender oil only had a significant attractive effect on male *T. flavus*, with an attraction rate of 71.88% (*p* = 0.03), suggesting that it could be a potential alternative to insect lures. In pot experiments, these EOs demonstrated sustained insecticidal effects and varied impacts on crops. Lavender oil only significantly affected the shoot length of soybeans (*Glycine max*), while mint oil did not significantly affect the growth of *G. max*. Finally, we preliminarily analyzed the chemical composition of the EOs to provide insights into their active components. These findings indicate that EOs have potential applications as natural agrochemicals, but further research on their mechanisms and application conditions is required.

## 1. Introduction

Lamiaceae, also known as the mint or sage family, is a large group of plants characterized by their distinctive labiate corolla. This family includes approximately 240 genera, with over 7000 species distributed globally [1,2]. Lamiaceae plants are renowned for their aromatic properties and medicinal values, making them widely used in food flavorings, cosmetics, and traditional medicine [3,4]. In recent years, they have also gained attention due to the insecticidal activities of their extracted essential oils (EOs) [5,6]. For example, *Mentha piperita* wild-type EO exhibits contact toxicity against *Aphis gossypii* and *Psylla Chinesis* [7]; patchouli oil repels *Aedes aegypti* and has toxic effects, making it a potential insecticide and repellent [8,9]; catnip oil repels subterranean termites [10] and *Stomoxys calcitrans* [11]; and lavender oil shows fumigant toxicity against *Orgyia trigotephras* [12]. Moreover, EOs have distinct advantages over chemical insecticides due to their high volatility and degradability, as well as their sensitivity to temperature, ultraviolet light, and sunlight, which result in lower persistence in the air and environment [13]. Given these characteristics, EOs are considered safer and more environmentally friendly than synthetic insecticides and pesticides, and they exhibit lower toxicity to mammals [14,15]. As natural insecticides, EOs have made some progress in commercialization, but their market penetration and application scope still have significant room for growth [16]. One key issue is that scholars have paid relatively little attention to the insecticidal effects of EOs.

*Thrips flavus*, belonging to the order Thysanoptera and the family Thripidae, is a worldwide pest widely distributed in Asian and European countries [17]. It can transmit plant viruses, posing a severe threat to agricultural yields [18,19]. In China, *T. flavus* is considered a major pest of flowering plants such as those in the Compositae and Leguminosae families in the northern regions [19,20,21]. Currently, the control of *T. flavus* primarily relies on chemical insecticides, but the excessive use of these chemicals leads to environmental pollution and the development of pest resistance [17]. Therefore, in recent years, EOs have become potential substitutes for chemical insecticides due to their environmental friendliness [22].

Some EOs, in addition to their insecticidal properties, can also serve as potential herbicides [23]. For example, the EO of the Lamiaceae plant *Thymus daenensis* reduces the germination rates of four types of weeds: *Amaranthus retroflexus*, *Avena fatua*, *Datura stramonium*, and *Lepidium sativum* [24]. EO extracted from *M. piperita* significantly inhibits the seed germination and early seedling development of the invasive weeds *Erigeron bonariensis* and *Araujia sericifera* [25]. Furthermore, some studies have shown that the herbicidal mechanism of EOs is involved in causing electrolyte leakage in plants, ultimately leading to plant death [26,27]. Although EOs have a certain degree of selectivity in their effects on plants [28], there is a concern about whether they may also harm crops. Currently, there is limited research on the impact of EOs on crop growth and development [1,29]. Therefore, in addition to assessing the effects of EOs on weed growth and development, we also evaluated their impact on crops. In this study, we selected soybean (*Glycine max*), a host crop of *T. flavus* [1], and corn (*Zea mays*), which is commonly intercropped with *G. max* [30], as well as two major weeds: barnyard grass (*Echinochloa oryzoides*) [31] and purslane (*Portulaca oleracea*) [32]. By evaluating the effects of EOs on both crops and weeds, this study aimed to understand their practical applications and to explore the potential development of herbicides while addressing the research gap regarding the impact of EOs on crop growth and development.

**Figure 1:**
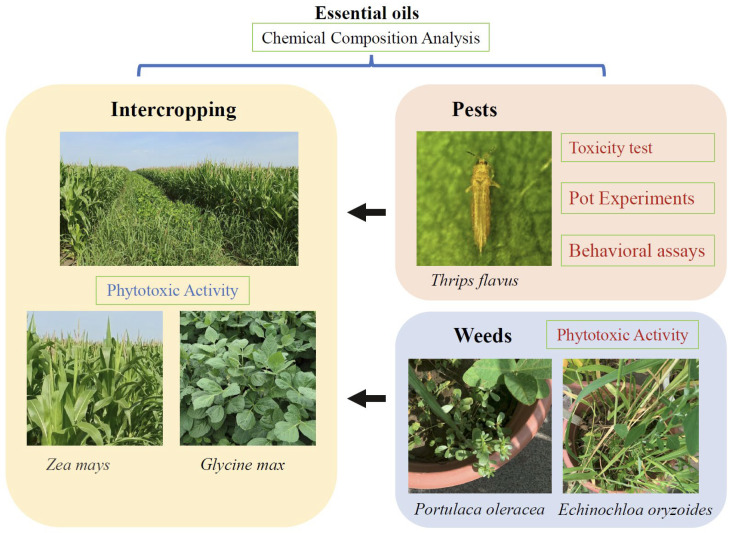
A comprehensive evaluation of four EOs. This study provides a systematic evaluation of the insecticidal effects and behavioral impacts of four EOs on *T. flavus*, utilizing indoor bioassays, olfactory behavior response tests, and a chemical component analysis. Moreover, it examines the effects of these EOs on crops (*G. max* and *Z. mays*) and weeds (*E. oryzoides* and *P. oleracea*). Finally, the compositional components of the four Lamiaceae EOs are analyzed.

This study aimed to systematically evaluate the insecticidal effects and behavioral impacts of four Lamiaceae EOs on *T. flavus* through indoor bioassays, olfactory behavior response tests, and a chemical component analysis of the plant EOs. Additionally, we assessed the effects of these EOs on crops and weeds to provide theoretical support for the development of commercial insecticides and herbicides derived from EOs. This research helps to understand the effectiveness of EOs in practical applications. Finally, we also analyzed the compositional components of the EOs to provide insights into their active ingredients (Figure 1).

## 2. Results

### 2.1. Toxicity of EO on T. flavus in Lab

Patchouli oil showed the strongest toxicity, with the lowest LC_50_ value of 0.31 mg/mL and a 95% confidence interval that did not overlap with the others. Next were catnip oil and lavender oil, with LC_50_ values of 0.33 mg/mL and 0.36 mg/mL, respectively. Mint oil had the highest LC_50_ value at 0.52 mg/mL, and its 95% confidence interval did not overlap with that of the other three EOs, indicating that it had significantly lower toxicity. The positive control, 45% malathion EC, exhibited higher toxicity to *T. flavus* than to the four EOs, with an LC_50_ of 0.0127 mg/mL (Table 1).

### 2.2. Toxicity of EOs to T. flavus in Pots

**Figure 2:**
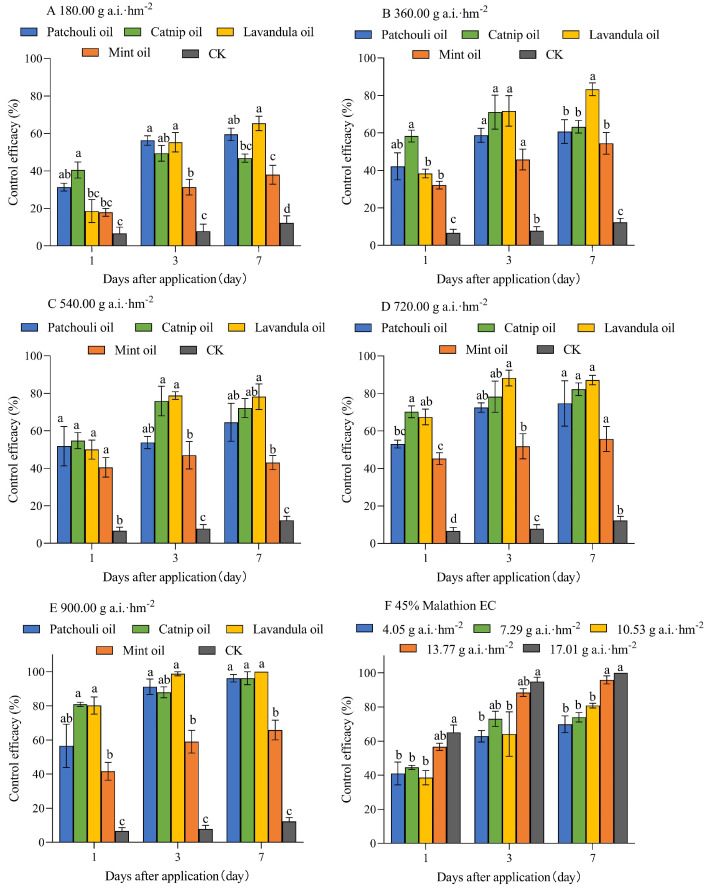
The pot experiments of the four plant EOs against *T. flavus* at different concentrations. (**A**) pot experiments of the four plant EOs at 180.00 g a.i.·hm^−2^; (**B**) pot experiments of the four plant EOs at 360.00 g a.i.·hm^−2^; (**C**) pot experiments of the four plant EOs at 540.00 g a.i.·hm^−2^; (**D**) pot experiments of the four plant EOs at 720.00 g a.i.·hm^−2^; (**E**) pot experiments of the four plant EOs at 900.00 g a.i.·hm^−2^; (**F**) pot experiments of 45% malathion EC. Different lowercase letters indicate significant differences among the efficacies of the four oils (*p* < 0.05).

The pot experiments of the four EOs at different concentrations showed significant differences over time after application (Figure 2). At a concentration of 180.00 g a.i.·hm^−2^, 7 days after application, the control efficacy of lavender oil was the highest at 65.38 ± 3.85%, significantly higher than that of catnip oil and mint oil (*F* = 37.171, *p* < 0.001, Figure 2A). At a concentration of 360.00 g a.i.·hm^−2^, 7 days after application, the control efficacy of lavender oil was the highest at 83.33 ± 3.39%, significantly higher than that of patchouli oil and mint oil (*F* = 35.265, *p* < 0.001, Figure 2B). At a concentration of 540.00 g a.i.·hm^−2^, 3 days after application, the control efficacy of catnip oil and lavender oil was 72.16 ± 5.06% and 78.2 ± 6.79%, respectively, significantly higher than that of mint oil (*F* = 31.043, *p* < 0.001, Figure 2C). At a concentration of 720.00 g a.i.·hm^−2^, 3 days after application, the control efficacy of lavender oil was the highest at 88.23 ± 4.24%, significantly higher than that of mint oil (*F* = 28.600, *p* < 0.001, Figure 2D). At a concentration of 900.00 g a.i.·hm^−2^, 7 days after application, the control efficacy of patchouli oil, catnip oil, and lavender oil was 96.2 ± 2.19%, 96.2 ± 3.8%, and 100%, respectively, significantly higher than that of mint oil (*F* = 51.068, *p* < 0.001, Figure 2E).

### 2.3. Behavioral Effects of EOs on T. flavus

**Figure 3:**
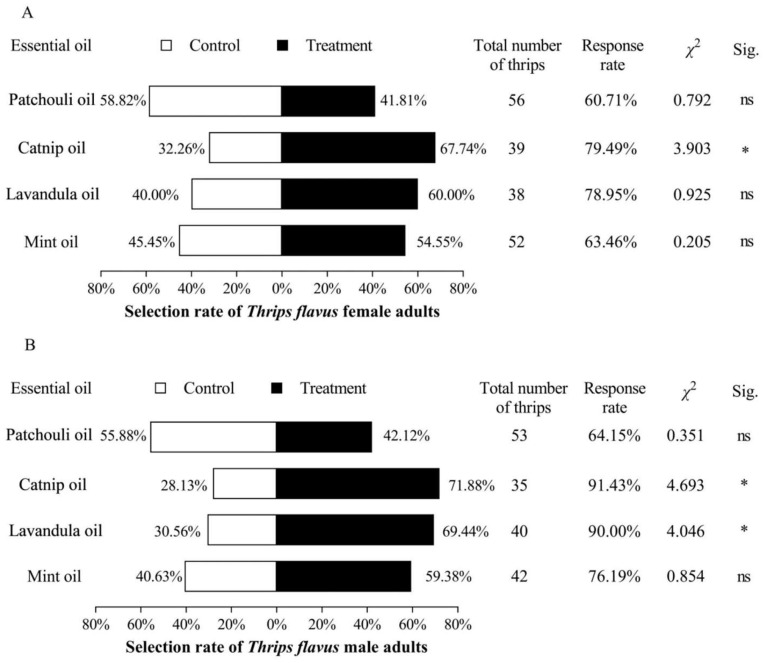
The olfactory behavior response of *T. flavus* to four plant EOs. (**A**) The olfactory behavior response of female adult *T. flavus* to the four EOs; (**B**) the olfactory behavior response of male adult *T. flavus* to the four EOs. “ns” indicates no significant difference between the control and treatment groups. “*” indicates a significant difference between the control and treatment groups (*p* < 0.05).

Catnip oil significantly attracted both male and female adult *T. flavus*, with attraction rates of 71.88% (*χ*^2^ = 4.693, *p* = 0.030) and 67.74% (*χ*^2^ = 3.903, *p* = 0.048), respectively. Lavandula oil significantly attracted male *T. flavus* only, with an attraction rate of 69.44% (*χ*^2^ = 4.064, *p* = 0.044), but it did not significantly attract female *T. flavus*. The remaining EOs did not have a significant effect on either male or female adult *T. flavus* (Figure 3).

### 2.4. Effects of Essential Oils on Plants

**Figure 4:**
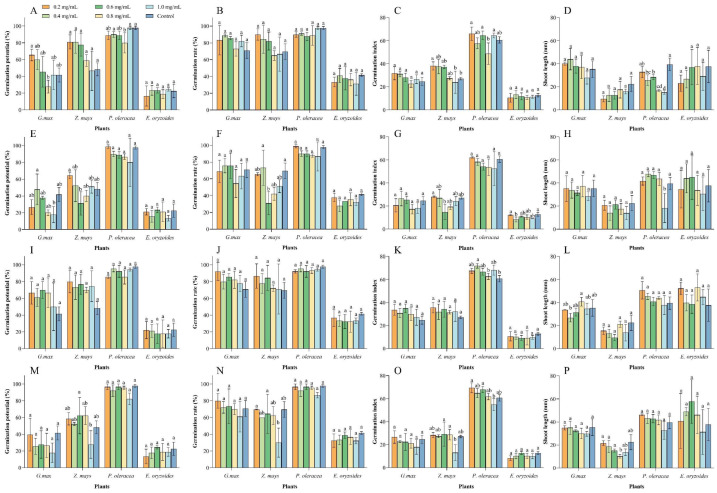
The phytotoxicity of the four essential oils on four plant species. (**A**–**D**) present the germination potential, germination rate, germination index, and radicle length of the four plants after treatment with patchouli essential oil. (**E**–**H**) correspond to the germination potential, germination rate, germination index, and radicle length of the four plants after treatment with nepeta essential oil. (**I**–**L**) show the germination potential, germination rate, germination index, and radicle length of the four plants after treatment with lavender essential oil. (**M**–**P**) indicate the germination potential, germination rate, germination index, and radicle length of the four plants after treatment with mint essential oil. Different lowercase letters indicate significant differences (*p* < 0.05) among the indices at different concentrations.

After treatment with different concentrations of the four EOs, there were significant differences in the germination potential, germination rate, germination index, and shoot length of the four plants. Patchouli oil had a significant impact on the germination potential of *G. max* (*F* = 3.549, *p* = 0.034), the germination index of *Z. mays* (*F* = 4.631, *p* = 0.014), the germination potential of *P. oleracea* (*F* = 4.641, *p* = 0.014), and the germination index of *P. oleracea* (*F* = 4.407, *p* = 0.016) (Figure 4A–D). Catnip oil showed significant differences in the germination potential of *G. max* (*F* = 4.483, *p* = 0.015), the germination index of *E. oryzoides* (*F* = 4.662, *p* = 0.013), and the shoot length of *P. oleracea* (*F* = 8.614, *p* = 0.001). This EO also had a significant effect on the germination rate (*F* = 5.645, *p* = 0.007), germination potential (*F* = 3.183, *p* = 0.047), and germination index (*F* = 4.089, *p* = 0.021) of *Z. mays* (Figure 4E–H). Lavender oil had a significant effect on the shoot length of *G. max*, where the shoot length at 0.8 mg/mL was significantly higher than at 0.4 mg/mL (*F* = 3.353, *p* = 0.04), but it did not have a significant impact on the other indicators of the four plants (Figure 4I–L). Mint oil had a significant effect on the germination index of *P. oleracea* (*F* = 6.198, *p* = 0.005); it also had a significant effect on all four indicators of *Z. mays*, where the shoot length at 0.8 mg/mL was significantly lower than that of the CK and at 0.2 mg/mL (*F* = 4.662, *p* = 0.013) (Figure 4M–P).

### 2.5. Chemical Composition of Four EOs

Patchouli oil contains 23 compounds, including terpenes, alcohols, and aldehydes, as well as derivatives of cyclobutane and naphthalene. The most abundant compound is patchoulol, with a relative content of 29.54%, followed by *β*-humulene, *α*-guaiene, *α*-bulnesene, and *α*-amorphene, with relative contents of 16.33%, 12.43%, 9.59%, and 8.36%, respectively (Table 2).

Catnip oil contains seven compounds, including terpenes, phenols, aldehydes, and derivatives of benzene. Limonene has the highest relative content at 51.31%, followed by o-cymene and 2-propenal, with relative contents of 20.32% and 12.90%, respectively (Table 3).

Lavender oil contains 17 compounds, including terpenes, alcohols, and esters. Linalyl acetate has the highest content at 17.90%, followed by terpinyl acetate at 16.66%, while octylmethacrylat has the lowest relative content at only 0.33% (Table 4).

Mint oil contains 19 compounds, including terpenes, alcohols, ketones, and esters. Menthol has the highest content at 27.54%, followed by isomenthone at 17.81% and limonene at 8.21% (Table 5).

## 3. Discussion

In recent years, with the growing awareness of environmental protection and food safety, people have increasingly focused on plant-derived insecticides such as EOs, which are considered safer and more environmentally friendly than synthetic pesticides [14,15]. These EOs have been proven effective in managing agricultural pests [1]. In this study, we measured the toxicity of four EOs to *T. flavus*. Patchouli oil, catnip oil, and lavender oil all showed good insecticidal activity, with patchouli oil being the most potent, followed by catnip oil and lavender oil, with LC_50_ values of 0.31, 0.33, and 0.36 mg/mL, respectively. These three EOs were significantly more toxic to *T. flavus* than mint oil. Compared to previous studies, these EOs showed higher toxicity towards *T. flavus* [19]. It has been reported that the fumigant toxicity of *Mentha pulegium* and *Thymus mastichina* EOs to *Frankliniella occidentalis* had LC_50_ values of 3.1 and 3.6 mg/L, respectively [33]; oregano oil and savory oil showed high insecticidal activity against *Plodia interpunctella* and *Ephestia kuehniella*, reaching 100% mortality within 24 h at concentrations of 9 µL/L air (for *P. interpunctella*) and 25 µL/L air (for *E. kuehniella*) [34]. The difference in toxicity in our study compared to these may be due to different testing methods or insect species. While toxicity tests are often conducted under laboratory conditions, we simulated natural conditions in pot experiments [1]. In the pot experiment analysis, we found that patchouli oil, lavender oil, and catnip oil were consistent with the bioassay results and showed good pest control effects; thus, they can be developed as potential insecticides. Additionally, although EOs are generally considered highly volatile [13] and require microencapsulation for sustained release [35], in our study, catnip oil still exhibited persistent insecticidal effects after seven days. This suggests that we may need to reassess the impact of the volatilization of some EOs on practical applications.

EOs play a crucial role in mediating interactions between plants and their environment [36]. Therefore, we evaluated the behavioral impacts of four EOs on *T. flavus*. Our results indicate that catnip oil and lavender oil have attractive effects on *T. flavus*, potentially enhancing insecticidal effects through attraction before killing [37]. Notably, in some studies, catnip oil could repel *A. aegypti* [38], *S. calcitrans* [11], and *Blattella germanica* [39]; this difference may be due to concentration variations. A study by Bedini et al. indirectly supports this finding, showing that the effects of six EOs on *Sitophilus zeamais* changed from attractive to repellent as the concentration increased [36]. Whether higher concentrations of catnip oil would repel *T. flavus* requires further investigation. In *Drosophila melanogaster* and *A. aegypti*, catnip oil activates the widely conserved chemosensory receptor TRPA1, and mutations in TRPA1 prevent these insects from being repelled by catnip oil. We speculate that the receptor for catnip oil in *T. flavus* is also TRPA1 [40]. Most studies on lavender oil have focused on its repellent effects, such as against Tabanidae and *Tribolium confusum* [41,42]. However, in our study, lavender oil was attractive to *T. flavus*. Interestingly, lavender oil only attracted male *T. flavus*, similar to the effects of sex pheromones on male *T. flavus* [43]. Therefore, lavender oil could serve as a potential substitute for more expensive pheromones.

In recent years, EOs have received considerable attention due to their herbicidal activity and environmental friendliness [23,24,25]. Some EOs cause electrolyte leakage leading to cell death in weeds [27,44]. Therefore, it is necessary to assess the safety of these EOs on crops. Our results show that all four EOs had varying negative impacts on crop growth and development, validating our concerns. We observed that the EOs had different effects on two crops and two weeds. Among them, lavender oil had the least impact on crops, only significantly affecting the shoot length of *G. max* but not impacting the weeds. Thus, lavender oil is not suitable as a potential herbicide but could be a potential insecticide. Mint oil did not adversely affect *G. max* growth but had significant effects on *Z. mays* at different growth stages, suggesting that it might be more suitable as a specific herbicide for *G. max* fields. Patchouli oil and catnip oil affected certain growth stages of crops. If these EOs are to be used as potential herbicides, care should be taken to avoid specific crops or growth stages, such as using mint oil only in *G. max* fields. Interestingly, patchouli oil and mint oil only affected the growth of *P. oleracea*. Therefore, patchouli oil and mint oil can be further developed as specific herbicides targeting *P. oleracea*.

Finally, we preliminarily analyzed the chemical compositions of the EOs to provide insights into their active components. The main chemical constituents identified in this study were consistent with those in other studies, but there were differences in their concentrations [45,46,47,48,49]. These differences depend on genetic, environmental, and processing factors [47]. The effects of EOs on insects and plants are mainly due to the presence of terpenoid compounds. In our study, we found a high content of terpenoids in the four EOs, including patchoulol, *β*-caryophyllene, *α*-guaiene, and *α*-bulnesene in patchouli oil; limonene and eugenol in catnip oil; *α*-pinene and camphor in lavandula oil; and menthol, menthone, and limonene in mint oil. For insects, some hypotheses support the notion that monoterpenes act on cytochrome P450, and certain terpenoids inhibit acetylcholinesterase activity [50]. For plants, some terpenes can inhibit seed germination and plant growth [51]. For example, eugenol in *Syzygium aromaticum* EO completely inhibits seed germination [52].

In summary, this study evaluated the bioactivity of four Lamiaceae EOs on *T. flavus*, crops, and weeds. We found that patchouli oil, catnip oil, and lavender oil had good insecticidal activity against *T. flavus*, with patchouli oil being the most potent. These EOs showed persistent insecticidal effects in pot experiments, indicating their potential as insecticides. Additionally, catnip oil and lavender oil had attractive effects on *T. flavus*, potentially enhancing insecticidal effects through attraction before killing. Regarding the impact on crop growth and development, lavender oil had the least impact on crops, while mint oil seemed more suitable as a specific herbicide for *G. max* fields. Finally, we analyzed the chemical compositions of the EOs to provide a basis for understanding their active components. Overall, these EOs have broad prospects as natural insecticides and herbicides, but further studies on the mechanisms and application conditions of the EOs are required in subsequent research.

## 4. Materials and Methods

### 4.1. Insects

The *T. flavus* individuals used in the experiment were collected from a *G. max* field in Changchun, Jilin Province (125°24′31″ E, 43°47′51″ N). They were captured using a sweep net and reared in insect cages on *G. max* for 2–3 days. Referring to the method by Han et al., the thrips species was identified under a stereomicroscope to ensure that it was *T. flavus* [53]. The rearing conditions were set to a temperature of 25 ± 1 °C, a relative humidity of 70% ± 5%, and a photoperiod of 16 h/8 h.

### 4.2. Essential Oils

The four Lamiaceae EOs tested were patchouli oil (*Pogostemon cablin*), catnip oil (*Nepeta cataria*), lavender oil (*Lavandula angustifolia*), and mint oil (*Mentha canadensis*). All EOs were provided by Ji’an Zhongxiang Natural Plants Co., Ltd., Ji’an, China, and they were extracted using the water–steam distillation method. The purity of the EOs was 98%.

### 4.3. Toxicity Test on T. flavus

To evaluate the toxicity of the EOs to the *T. flavus* adults, a feeding assay was performed. The method was based on oral toxicity with a few modifications [1]. The four EOs were pre-dissolved in acetone (Tianjin Xintong Fine Chemical Co. Ltd., Tianjin, China, purity 99.5%) and diluted with water to concentrations of 0.2 mg/mL, 0.4 mg/mL, 0.6 mg/mL, 0.8 mg/mL, and 1.0 mg/mL (acetone content was 0.5% for all solutions); 45% malathion (purchased from Hebei Jindelun Biochemical Technology Co. Ltd., Shijiazhuang, China; emulsifiable concentrate) served as the positive control. Fresh leaves of *G. max* (variety “Jinong 28”, provided by the Jilin Agricultural University), free from disease and insect damage and of uniform size, were selected, washed with distilled water, and air-dried. The leaves were then immersed in the test solution for 10 s, removed, and allowed to air dry. They were subsequently placed in 50 mL plastic centrifuge tubes containing moist filter paper. Water containing acetone (0.5% acetone content) served as the control. Thirty 3-day-old *T. flavus* adults were introduced into the test tubes for the experiment. Each treatment was replicated three times. After 24 h at room temperature, the mortality of *T. flavus* was checked, and the total numbers of insects and dead insects were recorded.

(1) $$M_{1}=\frac{ND}{NA}\times 100$$

(2) $$M_{2}=\frac{{MR}_{1}-{MR}_{2}}{1-{MR}_{2}}\times 100$$

where *M*_1_ is the mortality (%); *ND* is the number of dead thrips; *NA* is the total number of thrips treated; *M*_2_ is the corrected mortality (%); *MR*_1_ is the mortality rate of the treatment (%); and *MR*_2_ is the mortality rate of the blank control (%).

### 4.4. Pot Experiments

When the second trifoliate leaf emerged on the *G. max* plants, pots with uniform growth were selected, and one healthy *G. max* per pot was retained. No pesticides were sprayed on *G. max* prior to the experiment. Before conducting the experiments, each soybean pot was watered to ensure that the plants had sufficient moisture. Subsequently, the top of the pots was sealed with a cardboard sheet to prevent the *T. flavus* from falling into the soil after death and to minimize soil moisture loss. No further watering was conducted during the experiment. Based on the indoor bioassay results, the pesticide was applied at a rate equivalent to 900 L of solution per hectare (1 hm^2^). Each dose was replicated three times, with untreated controls (ddH_2_O) included. Using a spray bottle, the solution was applied uniformly, with 5 mL applied to each pot, and each plant was infested with 30 *T. flavus* adults. The pots were arranged with a 1 m spacing between treatments in a randomized block design. The number of dead thrips was observed and recorded 1 day, 3 days, and 7 days after spraying, and the efficacy in the pot trials was calculated according to the following formula:

(3) $$CE=\left(1-\frac{AT\times BC}{BT\times AC}\right)\times 100$$

where *CE* is the control efficacy (%); *AT* is the number of live thrips in the treated pots after treatment; *BC* is the number of live thrips in the control pots before treatment; *BT* is the number of live thrips in the treated pots before treatment; and *AC* is the number of live thrips in the control pots after treatment.

### 4.5. Behavioral Assays

The olfactory behavior responses of *T. flavus* to the plant EOs were tested using a Y-tube olfactometer [1]. The EOs were diluted to a concentration of 1.0 mg/mL in acetone. A 1 µL aliquot of the oil solution was applied to a 1 cm × 1 cm filter paper strip and placed in the odor-source bottle. Acetone alone was used as the control. The stem of the Y-tube was connected to a vacuum pump, and air was blown into the main arm of the Y-tube glass apparatus at a flow rate of 0.5 L/min. To maintain consistent lighting, the Y-tube was placed inside a light box during the experiment, with an average light intensity of 7800 to 8000 lx. Each adult thrips was observed for 5 min. When the thrips crossed the midpoint of either arm of the Y-tube, it was considered to have chosen the reagent on that side, and the attraction and repellency rates were calculated. If no choice was made within 5 min, it was recorded as no selection. Thirty male and thirty female adult thrips were tested for each EO. The sex of the thrips was determined by examining the shape of their genitalia under a stereomicroscope. The *T. flavus* individuals used in the behavioral assays were all 3 days old. The ovipositor of the female *T. flavus* consists of two pairs of sclerotized, toothed valves, with grooves between them that serve as the passage for eggs. The aedeagus of the male *T. flavus* is a slender, backward-curving structure that extends slightly upward, featuring either a pointed or blunt tip [21]. The numbers of thrips choosing the treatment and control groups were recorded, and the attraction and repellency rates were calculated using Formulas (1) and (2), respectively:

(4) $$S=\frac{CE}{Rs}\times 100$$

(5) $$R=\frac{CC}{Rs}\times 100$$

where *S* is the attraction rate (%); *CE* is the number of insects choosing the EO; *Rs* is the number of insects with a response; *R* is the repellency rate (%); and *CC* is the number of insects choosing the control.

### 4.6. Phytotoxic Activity of EOs

A plant toxicity test was conducted following the method described by Kasrati et al. [54]. The four EOs were mixed with Tween-80 (Sinopharm Chemical Reagent Co., Ltd., Shanghai, China) at a ratio of 1:1 (*v*/*v*). The mixtures were then diluted with distilled water to five concentrations: 0.2, 0.4, 0.6, 0.8, and 1.0 mg/mL. Distilled water containing Tween-80 was used as the control. Plant toxicity was tested on four plants: *Glycine max* (variety “Jinong 28”, provided by the Jilin Agricultural University), *Z. mays* (corn variety JS416, purchased from Jilin Huawei Agricultural Science and Technology Development Co., Ltd., Jilin, China), *E. oryzoides*, and *P. oleracea*. The seeds of these plants were disinfected with 1% sodium hypochlorite (Guangdong Wenlong Biotechnology Co., Ltd., Yangjiang, China); then, they were rinsed three times with distilled water and filtered. The disinfected seeds were placed in glass Petri dishes. Thirty seeds were added to each dish, and the EO solutions were applied, with three replicates for each concentration. One layer of filter paper was placed above and below each layer of seeds. The filter papers were sprayed daily with the corresponding concentrations of EO solutions to keep them moist. Seed germination was considered to occur when the radicle broke through the seed coat and reached a length of 1 mm. All treatments were incubated in complete darkness at a temperature of 25 ± 1 °C and a relative humidity of 70 ± 5% in a controlled growth chamber. On the 7th day, the shoot lengths (SLs) were measured using a vernier caliper. The germination rate, germination potential, and germination index were calculated.

The calculation formulas for the germination rate, germination potential, and germination index were as follows:

(6) $$GR=\frac{G_{1}}{G_{0}}\times 100$$

(7) $$GP=\frac{G_{2}}{G_{0}}\times 100$$

(8) $$GI=\sum \frac{Gt}{Dt}$$

where *GR* is the germination rate, *G*_1_ is the number of germinated seeds, *G*_0_ is the total number of seeds, *GP* is the germination potential, *G*_2_ is the number of seeds germinated by day 3, *GI* is the germination index, *Gt* is the number of seeds germinated on the day, and *Dt* is the number of days of germination.

### 4.7. Chemical Composition Analysis

The chemical components of the EOs were analyzed using gas chromatography–mass spectrometry (GC-MS) equipped with a DB-5 capillary column (30 m × 0.25 mm i.d., 0.25 µm). The injection mode and temperature program followed the methods described by Pei et al. [1]. Compound identification was performed using GC-MS software applications by making a comparison with the mass spectral libraries NIST 147 and NIST 27.

### 4.8. Statistics

The toxicity regression equation was fitted using DPS 13.50 [53] (Hangzhou Ruifeng Information Technology Co., Ltd., Hangzhou, China, http://www.dpsw.cn, accessed on 14 September 2024) to obtain the correlation coefficient, calculate the LC_50_ value, and determine the 95% confidence interval. The pot experiment results were subjected to arcsine square root transformation using IBM SPSS Statistics (version 23.0, International Business Machines Corporation, Armonk, NY, USA), and significant differences among treatments were compared using Tukey’s test in a one-way ANOVA. The results of the olfactory behavior response tests were analyzed using a chi-square test to compare the significant differences between the treatment and control groups. Graphs were created using GraphPad Prism 9.50 (GraphPad Software, Boston, MA, USA).

## Figures and Tables

**Table 1:** Toxicity of four EOs and malathion to *T. flavus*.

Number	EOs	Regression Equation	Correlation Coefficient	LC50 (mg/mL)	95% Confidence Interval	*χ* ^2^	*df*
1	Patchouli oil	*y* = 6.6940 + 3.3029*x*	0.96	0.31	0.22–0.38	2.29	3
2	Catnip oil	*y* = 6.8508 + 3.8827*x*	0.99	0.33	0.26–0.40	0.58	3
3	Lavandula oil	*y* = 6.3242 + 2.9614*x*	0.78	0.36	0.27–0.43	7.36	3
4	Mint oil	*y* = 6.1968 + 4.2378*x*	0.88	0.52	0.45–0.60	4.44	3
5	45% Malathion EC	*y* = 9.7959 + 2.5307*x*	0.94	0.0127	0.0068–0.0168	1.97	3

**Table 2:** Chemical composition of patchouli oil.

Number	Retention Index	Relative Percentage (%)	Name of Constituent
1	935	0.08	*α*-pinene
2	974	0.17	*β*-pinene
3	1340	0.14	*δ*-elemene
4	1387	3.22	*β*-patchoulene
5	1391	1.46	*β*-elemene
6	1415	1.08	thujopsen
7	1422	4.18	*β*-caryophyllene
8	1440	12.43	*α*-guaiene
9	1447	8.36	*α*-panasinsene
10	1456	1.21	*α*-bisabolene
11	1463	9.59	*α*-bulnesene
12	1471	0.63	caryophyllene
13	1478	1.05	alloaromadendrene
14	1489	0.83	*α*-guaiene
15	1500	5.55	longifolene
16	1506	16.33	*β*-humulene
17	1520	0.39	*β*-panasinsene
18	1528	0.14	cubebene
19	1551	0.97	isopatchoulane
20	1568	0.30	*α*-longipinene
21	1579	0.63	viridiflorol
22	1618	0.91	widdrol
23	1657	29.54	patchouli alcohol

**Table 3:** Chemical composition of catnip oil.

Number	Retention Index	Relative Percentage (%)	Name of Constituent
1	1013	20.32	o-cymene
2	1024	51.31	limonene
3	1051	2.65	*γ*-terpinene
4	1235	12.90	2-propenal
5	1332	10.45	eugenol
6	1418	1.85	*β*-caryophyllene
7	1454	0.51	*α*-caryophyllene

**Table 4:** Chemical composition of lavender oil.

Number	Retention Index	Relative Percentage (%)	Name of Constituent
1	934	7.24	*α*-pinene
2	1021	4.40	1,8-cineole
3	1086	8.01	3-Octanol
4	1126	7.94	camphor
5	1136	0.76	*β*-terpineol
6	1159	0.75	borneol
7	1171	0.45	terpinen-4-ol
8	1179	6.16	*α*-terpineol
9	1185	2.42	*γ*-terpineol
10	1246	17.90	linalyl acetate
11	1270	4.48	cyclohexanol
12	1274	9.81	bornyl acetate
13	1318	1.12	*β*-terpinyl acetate
14	1335	16.66	terpinyl acetate
15	1347	0.33	*octylmethacrylat*
16	1408	9.95	lignyl acetate
17	1420	0.80	*β*-caryophyllene

**Table 5:** Chemical composition of mint oil.

Number	Retention Index	Relative Percentage (%)	Name of Constituent
1	933	4.55	*α*-pinene
2	968	0.66	*α*-phellandrene
3	973	4.64	*β*-pinene
4	983	1.77	3-octanol
5	1023	8.21	limonene
6	1140	1.69	isopulegol
7	1143	17.81	isomenthone
8	1153	10.41	menthone
9	1161	6.73	neoisomenthol
10	1168	27.54	menthol
11	1176	1.26	isomenthol
12	1179	0.91	*α*-terpineol
13	1214	1.97	pulegone
14	1222	1.22	valeric acid 3-hexen-1-yl ester
15	1228	1.31	piperitone
16	1280	7.86	menthyl acetate
17	1387	0.24	*β*-bourbonene
18	1420	0.93	*β*-caryophyllene

## Data Availability

The data are contained within the article.
